# Supplementary material for: Cleaner fish are sensitive to what their partners can and cannot see
Source: Commun Biol. 2021 Sep 30;4:1127. doi: 10.1038/s42003-021-02584-2 (PMC8484626; doi:10.1038/s42003-021-02584-2)
Supplement: Supplementary file 2 — Supplementary Information [file 42003_2021_2584_MOESM2_ESM.pdf]

Supplementary online materials for *Cleaner fish are sensitive to what their partners can and cannot see*

Table of Contents

Figure S1: Individual data for Study 1 2

Figure S2: Flake items by round in Study 1 3

Table S1: Model output for Study 1 4

Figure S3: Punishment by flake in Study 1 5

Table S2: Model output for Study 2 6

Figure S4: Barrier choice in Study 2 by pair. 7

Table S3: Lengths and Widths 8

Figure S5: Barrier choice in Study 2 separated into four rather than two blocks of trials... 9

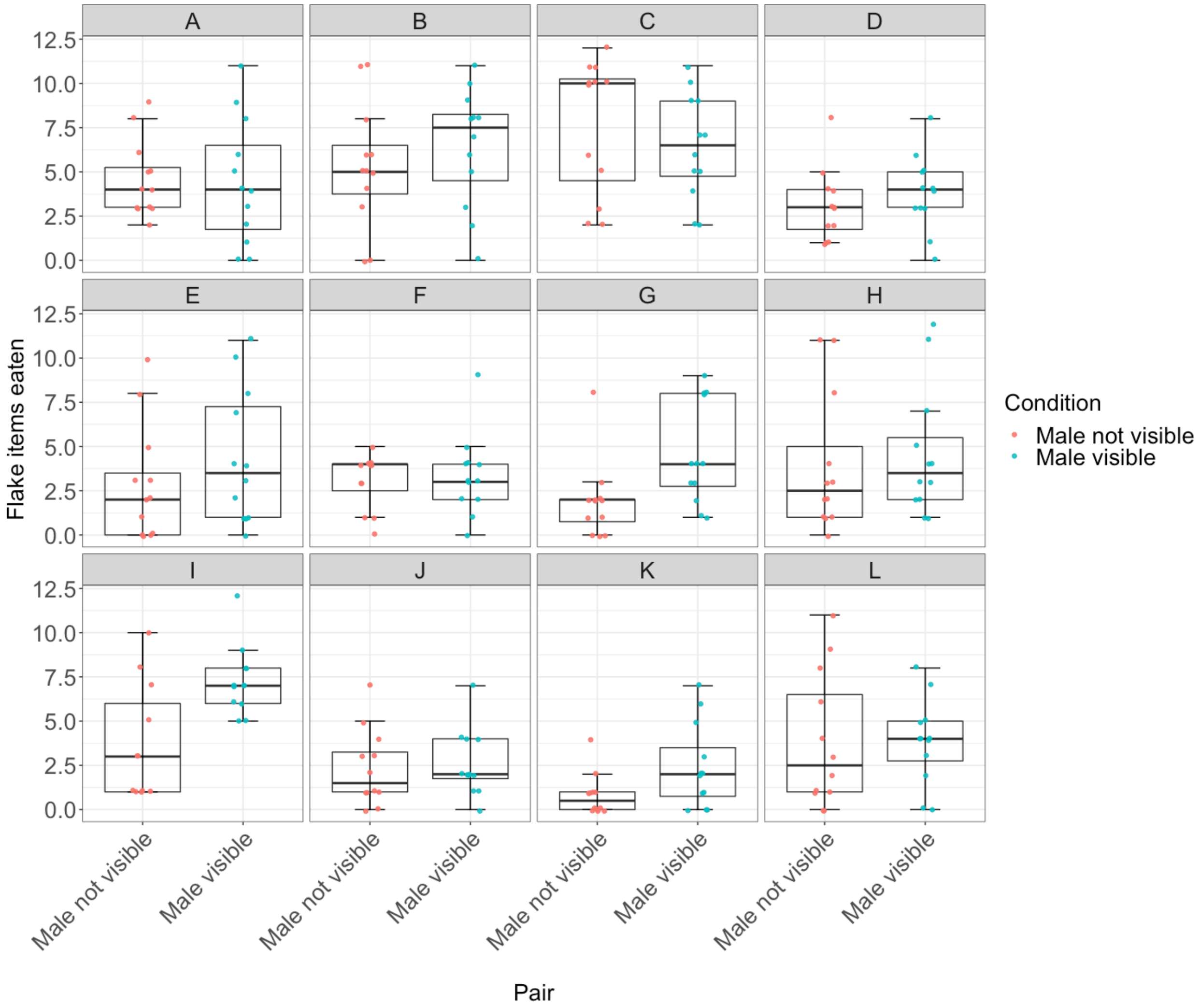

Figure S1: Individual data for Study 1

Boxplot showing flake items eaten by females across conditions along with raw data. Plot is faceted by pair identifier.

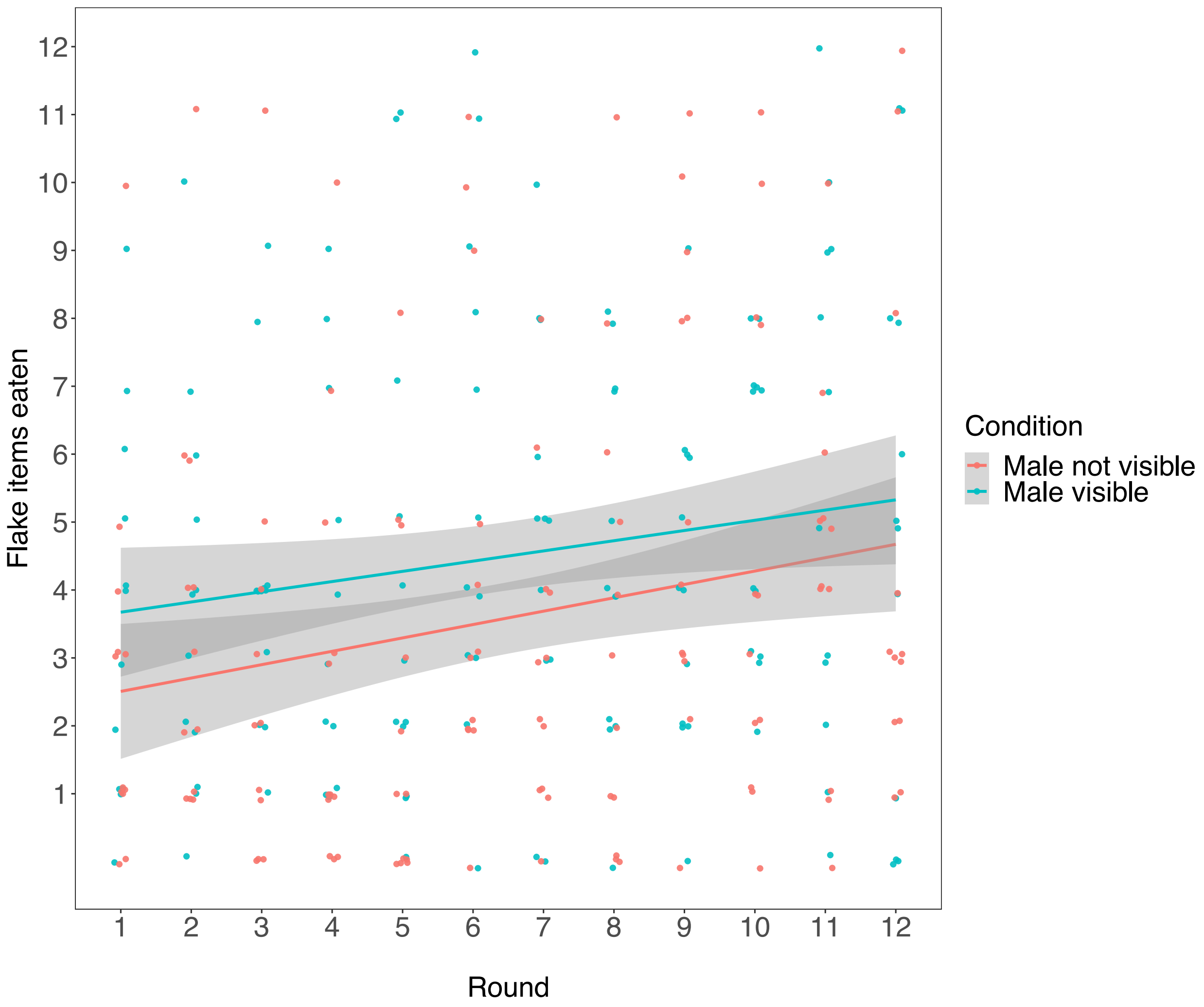

Figure S2: Flake items by round in Study 1

Plot showing number of flake items eaten by condition across round along with raw data.

Table S1: Model output for Study 1

Estimate and bootstrapped CIs (number of simulations = 1,000) of fixed effects in LMMs and GLMMs reported in the main text. Baseline for condition term = Male not visible. Table also shows goodness-of-fit statistics.

	**DV = flake items eaten**			**DV = punishment (binary)**		
	**Null**	**Full**	**Full with interaction**	**Null**	**Full**	**Reduced**
Intercept	**4.05 [3.16; 4.92]***	**2.51 [1.38; 3.56]***	**2.35 [1.08; 3.71]***	-0.65 [-1.48; 0.16]	-0.26 [-1.34; 0.82]	-0.31 [-1.33; 0.65]
Condition: Male visible		**0.95 [0.30; 1.59]***	1.25 [-0.06; 2.59]		0.04 [-0.92; 1.02]	0.16 [-0.41; 0.74]
Round		**0.09 [0.04; 0.13]***	**0.10 [0.03; 0.17]***		-0.00 [-0.04; 0.04]	-0.00 [-0.04; 0.04]
Condition x Round			-0.02 [-0.12; 0.07]			
Flake items eaten by female					-0.12 [-0.26; 0.02]	**-0.10 [-0.21; -0.00]***
Condition x Flake items eaten by female					0.03 [-0.18; 0.22]	
AIC	1453.89	1444.50	1450.48	335.70	339.05	337.16
BIC	1464.87	1462.80	1472.44	343.01	360.97	355.42
Log Likelihood	-723.94	-717.25	-719.24	-165.85	-163.53	-163.58
Number of trials	287	287	287	285	285	285
Number of pairs	12	12	12	12	12	12
Variance: Pair ID (Intercept)	1.94	1.95	1.94	1.64	1.60	1.62
Variance: Residual	8.44	7.92	7.95			

* **0 outside the confidence interval**

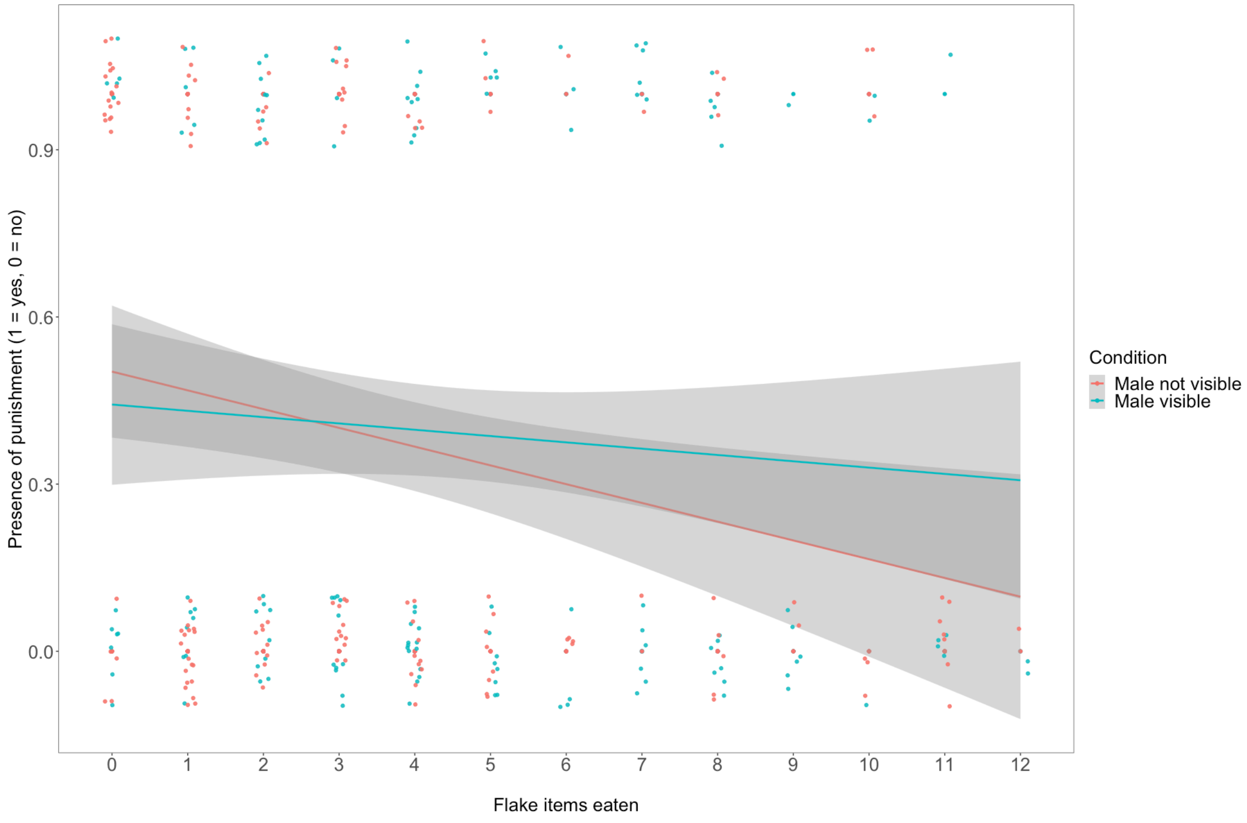

Figure S3: Punishment by flake in Study 1

Plot showing punishment (binary) by number of flake items eaten across condition along with raw data.

Table S2: Model output for Study 2

Estimate and bootstrapped CIs (number of simulations = 1,000) of fixed effects in LMMs and GLMMs reported in the main text. Baseline for condition term = Male not visible; block term = First block; barrier choice term = Transparent. Table also shows goodness-of-fit statistics.

	**DV = Barrier choice (1 = opaque)**			**DV = Punishment (binary)**			**DV = Flake items eaten by female**		
	**Null**	**Full**	**Full with interaction**	**Null**	**Full**	**Reduced**	**Null**	**Full**	**Block 1 only**
Intercept	0.10 [-0.13; 0.35]	-0.60 [-1.49; 0.20]	**-2.46 [-3.84; -1.41]***	**-1.62 [-2.58; -0.83]***	**-2.45 [-4.06; -1.40]***	**-2.28 [-3.52; -1.35]***	**1.44 [1.22; 1.67]***	**1.62 [1.31; 1.91]***	**1.57 [1.21; 1.95]***
Condition: Male visible		-0.01 [-0.56; 0.45]	**3.57 [2.10; 5.44]***		0.76 [-0.37; 2.06]	0.46 [-0.22; 1.14]		-0.23 [-0.55; 0.13]	-0.01 [-0.49; 0.49]
Block: Second block		0.47 [-0.02; 1.01]	**1.72 [1.03; 2.58]***						
Condition x Block			**-2.39 [-3.48; -1.46]***						
Barrier choice: Opaque					0.99 [-0.12; 2.40]	**0.72 [0.05; 1.49]***		-0.34 [-0.65; 0.01]	-0.30 [-0.85; 0.24]
Condition x Barrier choice					-0.50 [-2.03; 1.00]			0.46 [-0.03; 0.93]	0.38 [-0.37; 1.12]
AIC	367.95	368.29	348.16	255.93	254.94	253.44	739.87	747.34	394.94
BIC	375.10	382.58	366.02	263.07	272.80	267.73	750.57	768.73	412.19
Log Likelihood	-181.98	-180.14	-169.08	-125.96	-122.47	-122.72	-366.94	-367.67	-191.47
Number of trials	263	263	263	263	263	263	261	261	131
Number of pairs	11	11	11	11	11	11	11	11	11
Variance: Pair ID (Intercept)	0.00	0.00	0.00	1.60	1.69	1.67	0.10	0.10	0.13
Variance: Residual							0.92	0.91	1.00

* **0 outside the confidence interval**

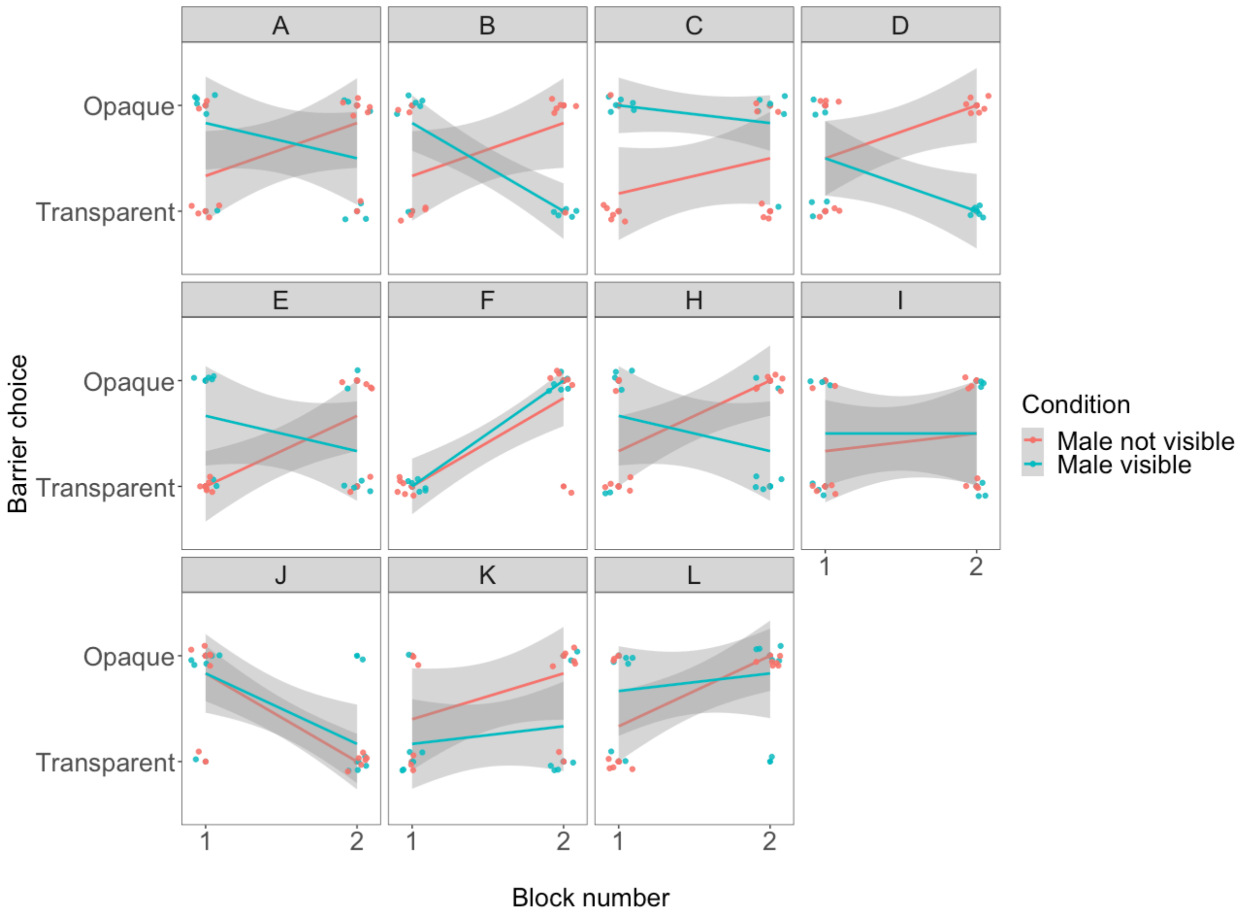

Figure S4: Barrier choice in Study 2 by pair.

Plot showing barrier choice (binary) by block number and condition. Data are shown separately for the 11 pairs of cleaner fish.

Table S3: Lengths and Widths

Lengths (L) and widths (W) of cleaner fish males and females tested in Studies 1 and 2.

Date	Couple	Male	Female	Where caught
18.03.2016	A	L = 9 cm	L = 7.5 cm	Motu Ahi
	A	W = 6.640 g	W = 3.670 g	
12.03.2016	B	L = 8.2 cm	L = 6.6 cm	Pharmacie
	B	W = 5.014 g	W = 2.636 g	
19.03.2016	C	L = 8.7 cm	L = 7.5 cm	Lolipop (pass side, right)
	C	W = 6.200 g	W = 3.420 g	
18.03.2016	D	L = 8.5 cm	L = 7 cm	Front Opunohu bay (shallow part on side)
	D	W = 5.185 g	W = 2.845 g	
13.03.2016	E	L = 9.5 cm	L = 8.2 cm	Pharmacie
	E	W = 7.7 g	W = 5.3 g	
25.03.2016	F	L = 8 cm	L = 7.4 cm	In front of waterfront bungalow
	F	W = 4.940 g	W = 4.120 g	
19.03.2016	G	L = 8.2 cm	L = 6.8 cm	Lolipop (pass side, right)
	G	W = 5.260 g	W = 3.100 g	
18.03.2016	H	L = 8.2 cm	L = 7 cm	Motu Ahi
	H	W = 4.730 g	W = 3.635 g	
15.03.2016	I	L = 8.6 cm	L = 7.6 cm	Motu Ahi
	I	W = 5.620 g	W = 4.690 g	
16.03.2016	J	L = 8.2 cm	L = 6.7 cm	Pinacle
	J	W = 4.760 g	W = 3.120 g	
19.03.2016	K	L = 7.8 cm	L = 7 cm	Lolipop (pass side, left)
	K	W = 3.870 g	W = 2.676 g	
20.03.2016	L	L = 8.5 cm	L = 8.5 cm	Lolipop black buoy on the left
	L	W = 6.825 g	W = 5.531 g	

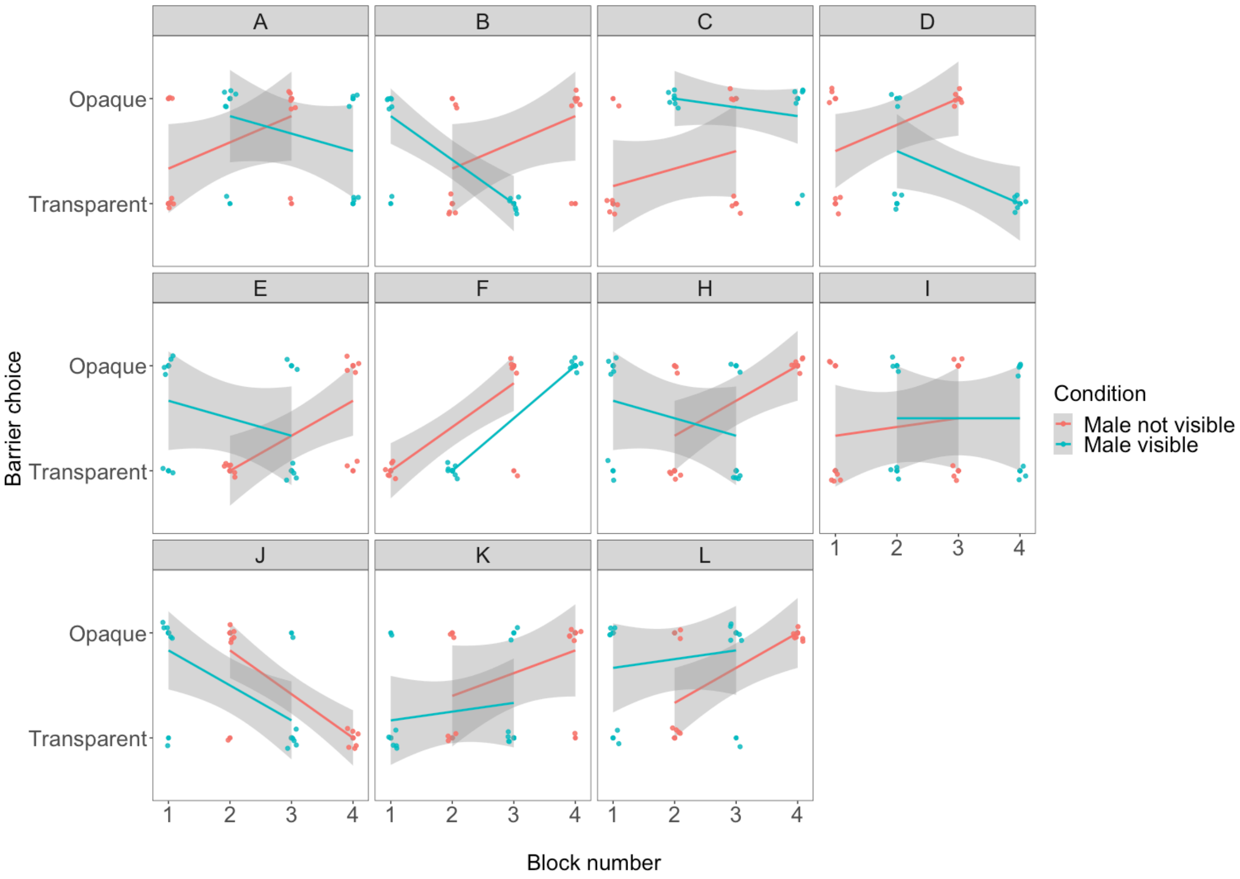

Figure S5: Barrier choice in Study 2 separated into four rather than two blocks of trials.

Plot showing barrier choice (binary) by block number and condition. Data are shown separately for the 11 pairs of cleaner fish. Note that in this plot "block" refers to blocks of 6 trials separated by condition. In other figures and in analyses block was fit as a two-level factor wherein block 1 contained data from the first presentation of each condition and block 2 contained data from the second presentation of each condition.
