# Supplementary material for: Cleaner fish are sensitive to what their partners can and cannot see
Source: Commun Biol. 2021 Sep 30;4:1127. doi: 10.1038/s42003-021-02584-2 (PMC8484626; doi:10.1038/s42003-021-02584-2)
Supplement: Supplementary file 3 — Description of Supplementary Files [file 42003_2021_2584_MOESM3_ESM.pdf]

Description of Additional Supplementary Files

File name: Supplementary Movie 1

Description: Video showing example trials from Study 1 and Study 2.

File name: Supplementary Data 1

Description: Raw data for Study 1.

File name: Supplementary Data 2

Description: Raw data for Study 2.
