# Supplementary material for: Cleaner fish are sensitive to what their partners can and cannot see
Source: Commun Biol. 2021 Sep 30;4:1127. doi: 10.1038/s42003-021-02584-2 (PMC8484626; doi:10.1038/s42003-021-02584-2)
Supplement: Supplementary file 7 — Reporting Summary [file 42003_2021_2584_MOESM7_ESM.pdf]

nature research

Corresponding author(s): Katherine McAuliffe

Last updated by author(s): Jun 16, 2021

Reporting Summary

Nature Research wishes to improve the reproducibility of the work that we publish. This form provides structure for consistency and transparency in reporting. For further information on Nature Research policies, see our Editorial Policies and the Editorial Policy Checklist.

Statistics

For all statistical analyses, confirm that the following items are present in the figure legend, table legend, main text, or Methods section.

n/a	Confirmed	
☐	☒	The exact sample size (*n*) for each experimental group/condition, given as a discrete number and unit of measurement
☐	☒	A statement on whether measurements were taken from distinct samples or whether the same sample was measured repeatedly
☐	☒	The statistical test(s) used AND whether they are one- or two-sided<br>*Only common tests should be described solely by name; describe more complex techniques in the Methods section.*
☐	☒	A description of all covariates tested
☒	☐	A description of any assumptions or corrections, such as tests of normality and adjustment for multiple comparisons
☐	☒	A full description of the statistical parameters including central tendency (e.g. means) or other basic estimates (e.g. regression coefficient) AND variation (e.g. standard deviation) or associated estimates of uncertainty (e.g. confidence intervals)
☐	☒	For null hypothesis testing, the test statistic (e.g. *F*, *t*, *r*) with confidence intervals, effect sizes, degrees of freedom and *P* value noted<br>*Give P values as exact values whenever suitable.*
☒	☐	For Bayesian analysis, information on the choice of priors and Markov chain Monte Carlo settings
☐	☒	For hierarchical and complex designs, identification of the appropriate level for tests and full reporting of outcomes
☐	☒	Estimates of effect sizes (e.g. Cohen's *d*, Pearson's *r*), indicating how they were calculated

Our web collection on statistics for biologists contains articles on many of the points above.

Software and code

Policy information about availability of computer code

Data collection	N/A
Data analysis	All statistical models were conducted in R version 3.6.3 (R Core Team, 2020). Mixed models were run using package lme4 (Bates, Mächler, Bolker, & Walker, 2015).

For manuscripts utilizing custom algorithms or software that are central to the research but not yet described in published literature, software must be made available to editors and reviewers. We strongly encourage code deposition in a community repository (e.g. GitHub). See the Nature Research guidelines for submitting code & software for further information.

Data

Policy information about availability of data

All manuscripts must include a data availability statement. This statement should provide the following information, where applicable:

- Accession codes, unique identifiers, or web links for publicly available datasets
- A list of figures that have associated raw data
- A description of any restrictions on data availability

Raw data have been provided and R code is available upon request.

Field-specific reporting

Ecological, evolutionary & environmental sciences study design

All studies must disclose on these points even when the disclosure is negative.

Study description	Quantitative experimental: repeated trials within subjects
Research sample	Pairs of cleaner fish (Labroides dimidiatus) captured and later released near Gump Field Station on the island of Mo'orea in French Polynesia.
Sampling strategy	Sample size was based on past experimental work with cleaner fish.
Data collection	Data were coded live during experiments and videos were checked for reliability.
Timing and spatial scale	Data were collected between March-April 2016.
Data exclusions	One of the pairs was not tested in Study 2 (pair G) because the female did not habituate to the testing set up. In study 1: flake information is missing for one trial in Study 1 and punishment information is missing for two trials (due to missing information in live coding). In Study 2: Flake information is missing for two trials due to missing information in live coding and choice data is missing for one trial due to no choice.
Reproducibility	We have endeavored to make our methods sufficiently detailed so as to facilitate replications.
Randomization	Subjects were not assigned to conditions as we employed a within-subject design. Design information (e.g., counterbalancing) is provided in text.
Blinding	We checked the reliability of our flake item DV in two ways. First, for the first six pairs tested, a researcher who was blind to condition counted the flake that had been eaten from the plates once they had been removed from the aquaria. Agreement between their counts and the live coding was very high (Pearson correlation, $r = 0.96$, $p < 0.001$, $N = 141$ trials).

Did the study involve field work? ☒ Yes ☐ No

Field work, collection and transport

Field conditions	Data were collected in March and April which is during the rainy season: hot and humid
Location	Gump Field Station on the island of Mo'orea in French Polynesia.
Access & import/export	The study was approved by Boston College and Yale University IACUCs (Yale: IACUC #2015-11627; BC: IACUC #2016-006-01).
Disturbance	We acknowledge that catching is a stressful event for the fish. Afterwards, the fishes adapt well to captive conditions and lose their shyness towards human experimenters. The cognitive experiments can be seen as behavioural enrichment. All fish were released at their site of capture.

Reporting for specific materials, systems and methods

We require information from authors about some types of materials, experimental systems and methods used in many studies. Here, indicate whether each material, system or method listed is relevant to your study. If you are not sure if a list item applies to your research, read the appropriate section before selecting a response.

Materials & experimental systems

n/a	Involved in the study	
☒	☐	Antibodies
☒	☐	Eukaryotic cell lines
☒	☐	Palaeontology and archaeology
☐	☒	Animals and other organisms
☒	☐	Human research participants
☒	☐	Clinical data
☒	☐	Dual use research of concern

Methods

n/a	Involved in the study	
☒	☐	ChIP-seq
☒	☐	Flow cytometry
☒	☐	MRI-based neuroimaging

Animals and other organisms

Policy information about studies involving animals; ARRIVE guidelines recommended for reporting animal research

Laboratory animals	N/A

Wild animals	Subjects were twelve pairs of cleaner fish that were caught with hand nets and a barrier net (2m long, 1.5m high of a mesh size of 5mm) in the surrounding water/reef by divers and brought into large round aquaria, built from plastic tank (with a diameter of 2.12m and 0.865m high) subdivided into four compartments with plexiglass panels. Therefore, we ended up with twelve sections of an approximate dimension of 100x30cm, each of which housed one male-female pair. The water reached 35cm high in each section. Each tank was covered with mesh to protect the fish from birds. Each section contained two PVC pipe tubes which served as refuges for the fish. Tanks were cleaned regularly by scraping off algae and siphoning out dirty water. Fish were fed ad libitum with mashed prawn smeared on plexiglass plates every non-experimental day. At the end of the field season, fish were returned to the reefs where they were initially caught.
Field-collected samples	Please see above
Ethics oversight	This research was approved by the Yale and Boston College IACUCs (Yale: IACUC #2015-11627; BC: IACUC #2016-006-01) as well as by the French Polynesian authorities responsible for the program 'Délégation à la Recherche.'

Note that full information on the approval of the study protocol must also be provided in the manuscript.
